# Quality of life of organic and conventional fruit growers in Poland - a pilot study

**DOI:** 10.3389/fpubh.2025.1729013

**Published:** 2025-12-29

**Authors:** Hubert Dobrowolski, Bartosz Szumigaj, Dariusz Włodarek, Renata Kazimierczak, Justyna Obidzińska, Ewa Rembiałkowska

**Affiliations:** 1 School of Medical & Health Sciences, VIZJA University, Warsaw, Poland; 2 Department of Functional and Organic Food, Institute of Human Nutrition Sciences, Warsaw University of Life Sciences (SGGW), Warsaw, Poland; 3 Department of Dietetics, Institute of Human Nutrition Sciences, Warsaw University of Life Sciences (SGGW), Warsaw, Poland

**Keywords:** conventional, fruit growers, health, organic, quality of life

## Abstract

Quality of life is a multifaceted issue that affects an individual’s overall perception of their life, depending on their expectations. It is primarily dictated by subjectively perceived physical health, mental health or social relationships. Our study aimed to examine the quality of life in a group of organic and conventional fruit growers. Fifty-three participants took part in the survey. The WHOQOL-BREF questionnaire was used to measure quality of life. The participants were also asked additional questions about their health situation, financial education, and the length of their farming experience. Body mass and height measurements were taken. Participants mostly rated their economic and health situations as good, with organic fruit growers rating their health situation as better than that of conventional fruit growers. Overall, participants scored 300 (231–388), with organic fruit growers scoring significantly higher than those in conventional production (*p* = 0.041). In the individual domains of quality of life, the participants mainly scored high. Organic fruit growers scored significantly more than conventional fruit growers in the aspect of social relationships (*p* = 0.047). Self-assessed health status compared to peers was correlated with physical health and psychological domains (*p* < 0.05). At the same time, the number of chronic diseases was associated with physical health and social relationship domains (*p* < 0.05). In conclusion, the quality of life among the group of fruit growers was at a reasonable level; however, the organic fruit growers scored better in self-rated health, overall quality of life, and the social relationships domain.

## 1 Introduction

Quality of life is a complex concept to define. It is a multifaceted construct that links together the various branches of life and the functioning of the individual as a whole. The World Health Organisation (WHO) defines Quality of Life as an individual’s perception of their position in life, in the context of the culture and value systems in which they live, and in relation to their goals, expectations, standards, and concerns (1). In the most general terms, it can be said that the concept of quality of life broadly encompasses how an individual perceives the “goodness” of multiple aspects of their life (2). This is, however, a critical issue, as it will be reflected in the overall sense of happiness. Quality of life encompasses several essential factors, including individual perceptions of health status, psychosocial status, and other aspects of life (3), which collectively influence the individual’s functioning in society and personal well-being. Mental function and perception, as well as spiritual and existential dimensions, and the broader quality of life, are also influenced by aspects of everyday life (4), demonstrating the significant contribution of spiritual values in shaping daily routines.

A key element of quality of life is the subjective assessment of one’s own health. Huber et al. (4) developed the concept of positive health, centred on terms such as ‘functioning, resilience and self-management’. This concept of health implies that a person with an illness can feel healthy. Thus, psychological functions and perceptions, as well as spiritual and existential dimensions, and the broader quality of life, all impact the dimensions of everyday life.

Numerous studies have examined the quality of life in various states. The vast majority, however, focused on diseases, conditions or disorders (5–17), or age, focusing on older people (18–20), which has a connection with physical or mental well-being and related perception of one’s own condition and capabilities. Some studies have also explored various topics, including religious beliefs (21–23) and social support issues (24–26). Few studies, however, examine the quality of life in relation to the job, which can significantly impact it due to its conditions, nature, or corresponding financial circumstances.

Farmers and orchardists caring for the farm have a specific working mode, which results in limited free time. It is not uncommon for them to spend whole days in their fields or orchards, with only brief breaks for rest or a meal. Those closest to them, such as coworkers and family, are also faced with the task of supporting farmers in their work and optimising the time they spend on both work and daily tasks and rest. Due to the nature of their work and the increased physical activity it entails, farmers and fruit growers may also exhibit different body compositions and weights, which can lead to the occurrence of multiple non-communicable diseases (NCDs). This means that both farmers and the immediate family will be characterised by a different way of functioning compared to the rest of society, which may be reflected in a different quality of life compared to the rest of society. However, there is a lack of Polish research on quality of life in this working group, and international research is severely limited.

The differences between organic and conventional farming are also worth highlighting. Regulated at European Union (EU) level by the European Commission Regulation (EU) 2018/848 on Organic Production and Labelling of Organic Product (27) and at national level by the law of 23 June 2022 on organic farming and production (28) the organic farming system takes a restrictive approach to the use of synthetic pesticides, the enrichment of feed, the use of excess medication and the possibility of using food additives in the product. From an orchardist’s perspective, however, this means more work and often requires a great deal of creativity, with even more limited time and a specific lifestyle. Few studies show differences in the health status of organic and conventional farmers. Nankongnab et al. (29) surveyed to investigate the differences in health status between conventional and organic farmers. They showed that conventional farmers were more likely to develop pesticide-related ailments (skin rash, blisters, headaches, dizziness). At the same time, their organic colleagues were more likely to complain about physical health-related factors such as pain, numbness, or weakness in the wrists/hands, fingers, upper back, hips, and ankles/feet, or hives, chest pain, mild fever, flatulence, and frequent urination (29). The mental health of organic farmers has also been the subject of several studies. A study by Cross et al. (30) indicated that although there were no significant differences in health between conventional and organic farmers, the second ones scored better on the Short Depression Happiness Scale, meaning that they were happier than their conventional counterparts, which was related to the greater range of duties performed at work and the lesser monotony of each day. Khan et al. (31) demonstrated that conventional farmers had significantly higher age-adjusted mean neurological symptoms compared to their organic counterparts. Finally, Brigance et al. (32) suggested in their study that there may be protective mental health factors unique to the organic farmers. They reported greater satisfaction with farming as a profession than their conventional counterparts, the benefits of being connected to the land, a sense of social and environmental responsibility, and involvement in community activities (32). On the other hand, a study by Soto Mas et al. (33) on health issues in organic farming reveals that although exposure to hazardous pesticides is lower among organic farmers, organic agriculture primarily relies on a few individuals performing multiple tasks related to cultivation, harvesting, and distribution. This may increase psychological and physical risk factors for organic farmers (33). All these issues imply a quality of life that has not yet been studied among orchardists. Moreover, comparisons of the quality of life between conventional and organic farmers and fruit growers are also lacking. It is therefore relevant to undertake a study of the quality of life among Polish fruit growers, taking into account the specific nature of their work, as well as the significant differences between conventional and organic orchard management.

Given the specific nature of the work of orchardists, as well as the significant differences between conventional and organic orchard management, and the lack of research on the quality of life in this population, our study aimed to determine the quality of life and some of its components in the population of Polish orchardists from the Mazowieckie and Łódzkie voivodships, together with a comparative analysis of the differences between organic and conventional orchardists. Our primary research hypothesis, based on several findings to date, is that organic fruit growers should experience a higher quality of life than conventional fruit growers.

## 2 Materials and methods

The study initially involved 54 participants, comprising 29 from an organic farm (17 orchardists and 12 partners) and 25 from a conventional farm (15 orchardists and 10 partners). The partners of the orchard keepers are characterized by similar lifestyles and physical activity, due to their assistance with the daily tasks of the fruit growers, as well as having a similar worldview on environmental issues and a similar diet, resulting from shared meals eaten as a family.

Due to the pilot nature of the study, convenience sampling was used. Recruitment was done with the help of organic fruit growers’ associations. Most of the fruit growers participating in the survey focused on apple cultivation, with a small percentage growing other fruits, which perfectly reflects the conditions of fruit growing in Poland. From the selected fruit growers who agreed to participate in the study, conventional fruit growers who lived in the nearest neighborhood were selected, which ensured similar environmental conditions and access to factors affecting quality of life (e.g., public transport, access to healthcare, availability of products in stores, etc.) in both groups studied, allowing for comparison.

Once the results were collected, the data were analyzed using a box plot. On this basis, extreme outliers were identified and a decision was made to remove observations (*n* = 1). The final analysis, therefore, included results from 53 participants, of which 29 were from the organic farm and 24 from the conventional farm (14 fruit growers and 10 partners).

Approval for the study was obtained from the Rector’s Committee on Ethics in Human Research at SGGW (Decision No. 7/RKE/2023/U of 30 January 2023). All participants in the study were informed about the study’s conduct, the measurements taken, the data collected, and the use of the information collected during the study. Written consent was obtained from each participant to participate in the study and in any research procedures. All questions and concerns of the study participants were addressed in detail and comprehensively by the research team conducting the measurements.

Participants had their body mass and height measured twice. The SECA 213 stadiometer was used to measure body height, while the Tanita BF-350 device was used to measure body weight. An arithmetic mean was drawn from the two measurements. If the results of the measurements differed significantly from each other, another (third) measurement was taken, and then two measurements close to each other were used for further analysis. Body mass was measured in light clothing, after which the estimated weight of this clothing was deducted. In contrast, body height was measured without shoes, with the head in the Frankfurt plane position.

A questionnaire created by the World Health Organisation, in an abbreviated version known as WHOQOL-BREF, was used to assess quality of life (34). This questionnaire is a proven, validated and accurate method of determining quality of life, recommended by the World Health Organisation. It consists of 26 questions, of which the first two questions deal with the general subjective perception of one’s quality of life (5-point Likert scale, from very bad to very good), as well as the subjective assessment of health status (5-point Likert scale, from very dissatisfied to very satisfied). In the remaining questions, respondents rate individual factors from the last 4 weeks that determine the quality of life in four domains: physical health (questions about pain, dependence on medical treatments, energy levels, mobility, sleep quality, ability to perform daily activities, and capacity for work), psychological well-being (questions about positive feelings, sense of meaning in life, thinking and concentration abilities, body image, self-esteem, and negative emotions), social relationships (questions about satisfaction with personal relationships, sexual life, and social support from friends and family), and environmental factors (questions about safety, environment quality and its impact on health, financial resources, information availability, leisure opportunities, home conditions, access to health services, and transport availability). All aspects are assessed on a 5-point Likert scale. The raw score in the assessment of the four domains was transformed into a 100-point scale, according to the instructions issued by the World Health Organization.

The respondents were also asked to state their current financial situation (choice from: very bad; bad; average; good; very good), their education (choice from: below secondary; secondary; higher) and their health status compared to their peers (choice from: worse; same; better; difficult to say). Participants in the survey also specified whether they had a chronic illness, how long they had been farming and, in the case of organic fruit growers, how long they had been farming organically.

Statistical analysis was performed using SPSS v. 28.0 software (IBM Corp.), R and RStudio (The R Foundation). The normality of the distribution was estimated using the Shapiro–Wilk test. Comparisons were made between groups of organic and conventional fruit growers, between the genders of the participants in the study, and between fruit growers and their partners using the Student’s *t*-test for normally distributed data and the Mann–Whitney U test for non-normally distributed data. The analysis of the correlation between the studied parameters was performed using Pearson’s test (for quantitative scales and normally distributed data) and Spearman’s test (for ordinal data or data with a distribution different from normal). The study’s defined significance level was set to *α* = 0.05.

## 3 Results

### 3.1 General characteristics of the study group

The study group had a mean age of 44 ± 8 years and a mean body mass and height of 83.7 ± 15.9 kg and 172.2 ± 8.9 cm, respectively. The mean BMI in the study group was 28.12 ± 4.4 kg/m^2^. No statistically significant differences were observed between the groups of organic and conventional fruit growers (*p* > 0.05, test *t*). Significant differences were found between the fruit growers and partner groups in terms of height (*p* = 0.001, test *t*), weight (*p* < 0.001, test *t*) and BMI (*p* = 0.022, test *t*), which was probably due to the overwhelming predominance of women in the partner group, compared to the group of fruit growers. No significant differences were observed between groups of fruit growers and partners in terms of age (*p* > 0.05, test *t*). The characteristics of the study group are presented in Table 1.

**Table 1:** Characteristics of the studṣy group.

Study group		Age [mean±SD median min-max]	Body mass [mean ±SD median min-max]	Body height [mean ±SD median min-max]	BMI [mean ±SD median min-max]
Organic	Overall	46 ± 8 45 33–63	83.8 ± 16.9 88 51.4–113.3	174 ± 8.1 172 161–189	27.57 ± 4.87 27.85 16.22–36.33
	Orchardist	46 ± 7 46 34–63	90.3 ± 12.4 91.7 65.1–113.3	177.2 ± 7.7 179.0 161–183	28.77 ± 3.76 27.99 22.26–36.33
	Partner	45 ± 9 44 33–62	74.6 ± 18.5 69.9 51.4–102.3	169.5 ± 6.5 168.5 161–183	25.87 ± 5.86 24.64 16.22–35.82
Conventional	Overall	42 ± 9 41 29–63	83.6 ± 15.0 80.9 59.6–109.0	170.0 ± 9.4 168.5 154–192	28.79 ± 3.75 28.91 22.19–37.72
	Orchardist	44 ± 9 43 30–63	89.6 ± 15.5 94.9 64.1–109.0	172.8 ± 11.0 171.5 154–192	29.90 ± 3.73 29.62 22.19–37.72
	Partner	40 ± 8 39 29–57	75.1 ± 9.5 74.8 59.6–88	166.1 ± 4.7 166.0 161–174	27.24 ± 3.35 26.82 22.99–33.68
Overall	Overall	44 ± 8 44 29–63	83.7 ± 15.9 85.6 51.4–113.3	172.2 ± 8.9 170.0 154–192	28.12 ± 4.40 28.21 16.22–37.72
	Orchardist	45 ± 8 45 30–63	90.0 ± 13.6 91.7 64.1–113.3	175.2 ± 9.5 176.0 154–192	29.28 ± 3.73 28.65 22.19–37.72
	Partner	43 ± 9 43 29–62	74.8 ± 14.8 74.6 51.4–102.3	168 ± 5.9 167 161–183	26.49 ± 4.83 25.66 16.22–35.82

The study group primarily consisted of individuals with an average (*n* = 23) or good (*n* = 25) financial situation. Only four participants indicated a worse situation than the average. No statistically significant differences were observed between the groups of organic and conventional fruit growers (*p* > 0.05, U Mann–Whitney test). Participants in the study had a secondary education (*n* = 21) or higher (*n* = 28). No differences were observed between the groups as well (*p* > 0.05, U Mann–Whitney test). A comparison of education and financial situations is shown in Table 2.

**Table 2:** Characteristics of the financial situation, education and health status in the study group.

Study group	Financial situation			Education			Health status		
	Category	*N*	% of total	Category	*N*	% of total	Category	*N*	% of total
Organic	Very bad	1	1.9	Below secondary	1	1.9	Difficult to say	7	13.2
	Bad	2	3.8	Secondary	10	18.9	Worse than peers	1	1.9
	Neither good nor bad	11	20.8	Higher	18	34.0	Same as peers	2	3.8
	Good	14	26.4				Better than peers	19	35.8
	Very good	1	1.9						
Conventional	Very bad	0	0	Below secondary	4	7.5	Difficult to say	6	11.3
	Bad	1	1.9	Secondary	10	18.9	Worse than peers	3	5.7
	Neither good nor bad	12	22.6	Higher	10	18.9	Same as peers	15	28.3
	Good	11	20.8				Better than peers	0	0
	Very good	0	0						
*p*-value**	0.797			0.085			<0.001*		

*Those who answered “difficult to say” were excluded from the analysis.

**U Mann–Whitney test.Bold values indicate statistical significance.

Table 2 also presents the self-assessment of the health status of the study participants. In the study group, the majority of participants (*n* = 17) reported that their health status was the same as or better than that of their peers. In contrast, organic fruit growers rated their health status significantly better than conventional fruit growers (*p* < 0.001, U Mann–Whitney test). Those who were unable to assess their health status (*n* = 13) were excluded from this comparison. The vast majority (*n* = 40) of participants did not have chronic diseases or had only one disease (*n* = 10). Four participants had more than one disease. There were no differences between the organic and conventional fruit grower groups in the incidence of chronic diseases (*p* > 0.05, U Mann–Whitney test).

### 3.2 Overall subjective quality of life and general health

When evaluating their overall quality of life, survey participants indicated responses as ‘neither good nor bad’ (*n* = 5), ‘good’ (*n* = 39) or ‘very good’ (*n* = 9). On average, participants scored 4.08 ± 0.5 (median, 4.0) on this question. There were no statistically significant differences between the group of organic and conventional fruit growers (*p* > 0.05, U Mann–Whitney test). There were also no differences in overall quality of life between the group of fruit growers and partners (*p* = 0.852, U Mann–Whitney test).

**Figure 1:**
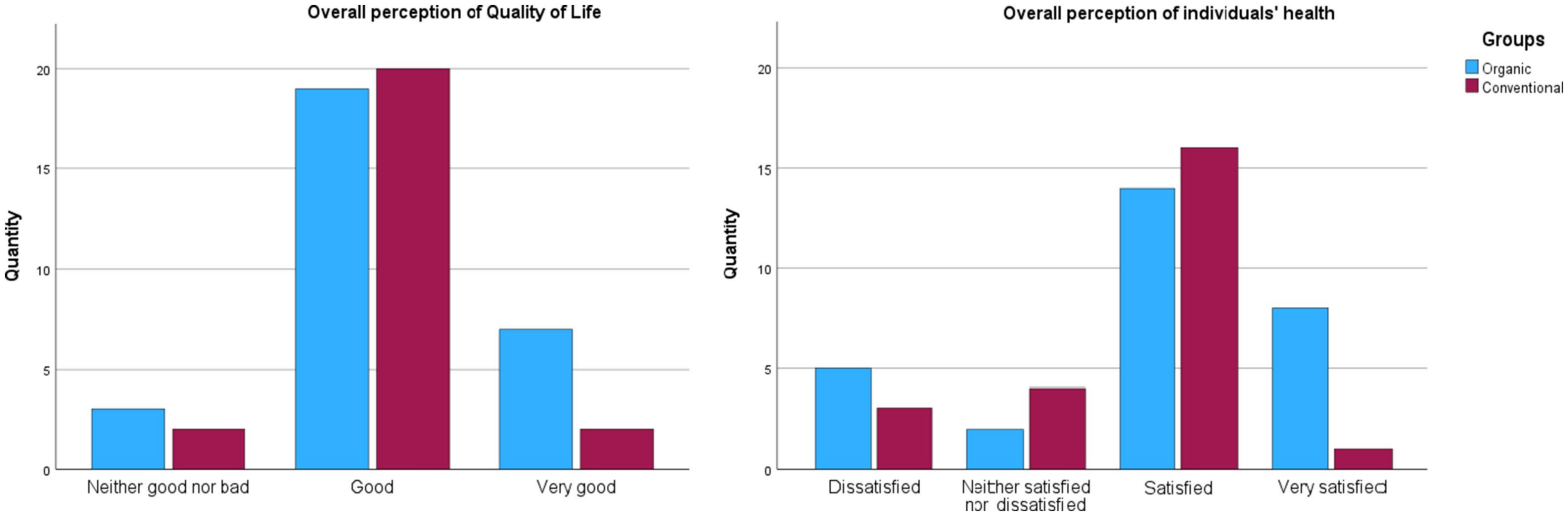
Subjective perceptions of quality of life and health by organic and conventional fruit growers using the WHOQOL-BREF questions.

When describing their subjective satisfaction with their health, participants indicated most often that they were ‘satisfied’ with their health (*n* = 30), but also ‘dissatisfied’ (*n* = 8), ‘neither satisfied nor dissatisfied’ (*n* = 6) or ‘very satisfied’ (*n* = 9). The study group scored an average of 3.75 ± 0.9 (median, 4.0) on this question. Similarly, in this case, there were no statistically significant differences between the organic and conventional fruit grower groups (*p* > 0.05, U Mann–Whitney test). Also, there were no differences in quality of life between the group of fruit growers and partners (*p* = 0.865, U Mann–Whitney test). Figure 1 shows the comparison in both questions between the groups of organic and conventional fruit growers (Figure 1).

A correlation was found between subjective assessment of quality of life and subjective satisfaction with one’s health status (*p* < 0.001, rho = 0.522, Spearman test). There was also a correlation between subjective quality of life and subjective health status and the number of scores obtained with the WHOQOL-BREF questionnaire (*p* < 0.001, rho = 0.591, Spearman test for overall quality of life; *p* < 0.001, rho = 0.506, Spearman test for subjective satisfaction with health status).

### 3.3 Quality of life overall and by domains

Table 3 presents the results of the quality of life assessment, including overall scores and scores by domain. Examination using the WHOQOL-BREF questionnaire revealed a quality of life in the study group, with a median score of 300 (231–388 points), corresponding to a maximum possible score of 400.

**Table 3:** Quality of life in the study group.

Study group	Quality of life				
	Domain				Overall [mean±SD median min-max]
	Physical health [mean±SD median min-max]	Psychological [mean±SD median min-max]	Social relationship [mean±SD median min-max]	Environment [mean±SD median min-max]	
Overall	73.92 ± 14.02 75 31–100	77.81 ± 12.32 81 44–100	81.74 ± 12.15 81 50–100	71.62 ± 12.66 69 44–94	305.09 ± 41.26 300 231–388
Organic	76.24 ± 12.78 75 50–100	80.28 ± 13.68 81 44–100	84.48 ± 11.83 81 56–100	74.52 ± 13.38 75 44–94	315.52 ± 42.78 319 238–388
Conventional	71.13 ± 15.18 69 31–100	74.83 ± 9.91 75 56–100	78.42 ± 11.92 75 50–100	68.12 ± 11.02 69 44–88	292.50 ± 36.32 288 231–375
*p*-value*	0.189	0.072	**0.047**	0.071	**0.041**

*For the overall quality of life and the psychological, social relationship and environmental domains, the U Mann–Whitney test was used due to the distribution of the data deviating from normal. For the physical health domain, the Student’s *t*-test was used due to the distribution of the data following a normal distribution.Bold values indicate statistical significance.

**Figure 2:**
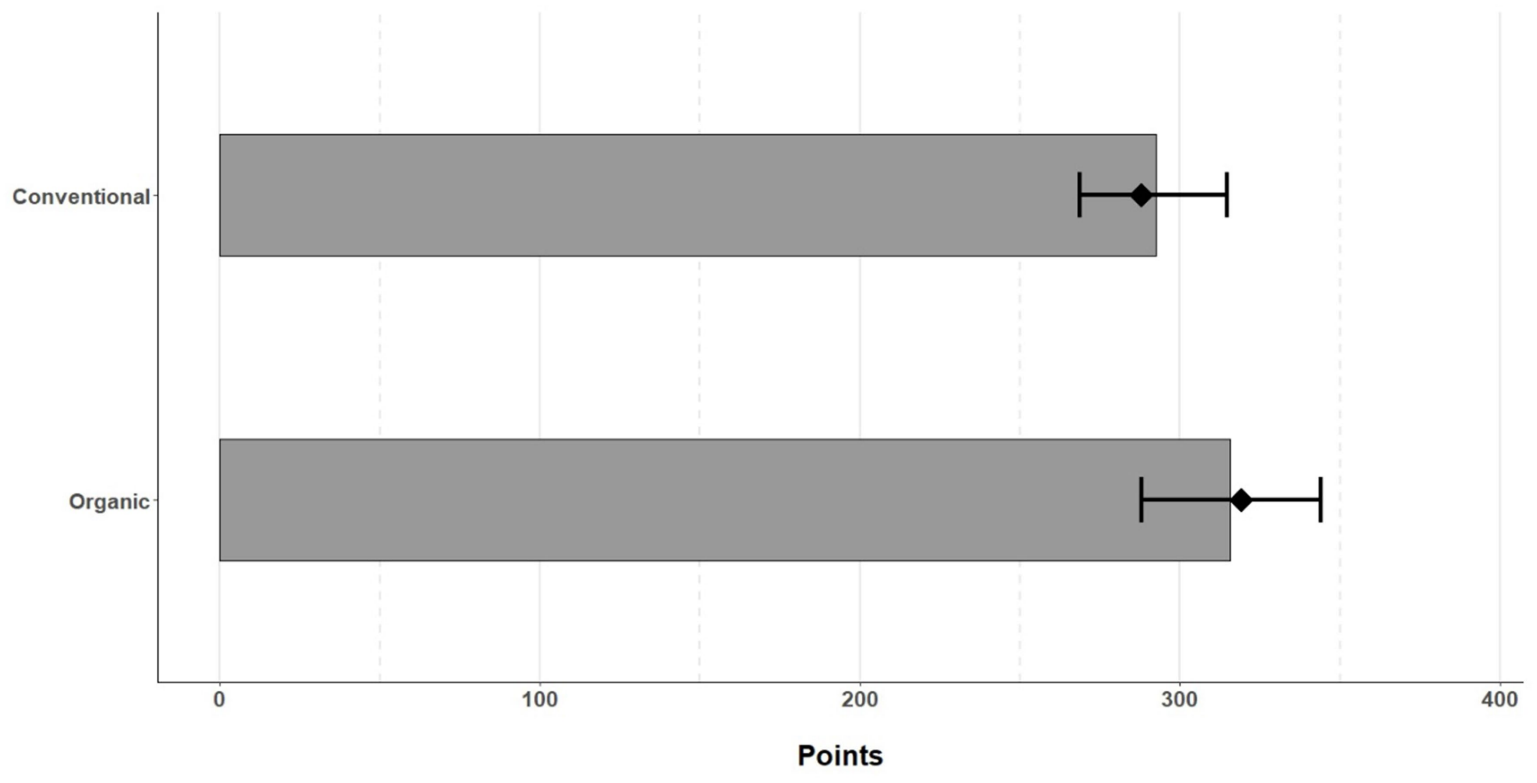
Differences in scores between the study groups.

In terms of overall quality of life, there was a significant difference between the groups, with organic orchardists scoring significantly higher (median 319; 238–388 points) compared to conventional orchardists (median 288; 231–375 points) (*p* = 0.041, U Mann–Whitney test). These differences are presented in the graph (Figure 2). No statistically significant differences were found between the group of fruit growers and partners (*p* > 0.05, U Mann–Whitney test). There were also no differences between men and women taking part in the study (*p* > 0.05, U Mann–Whitney test).

**Figure 3:**
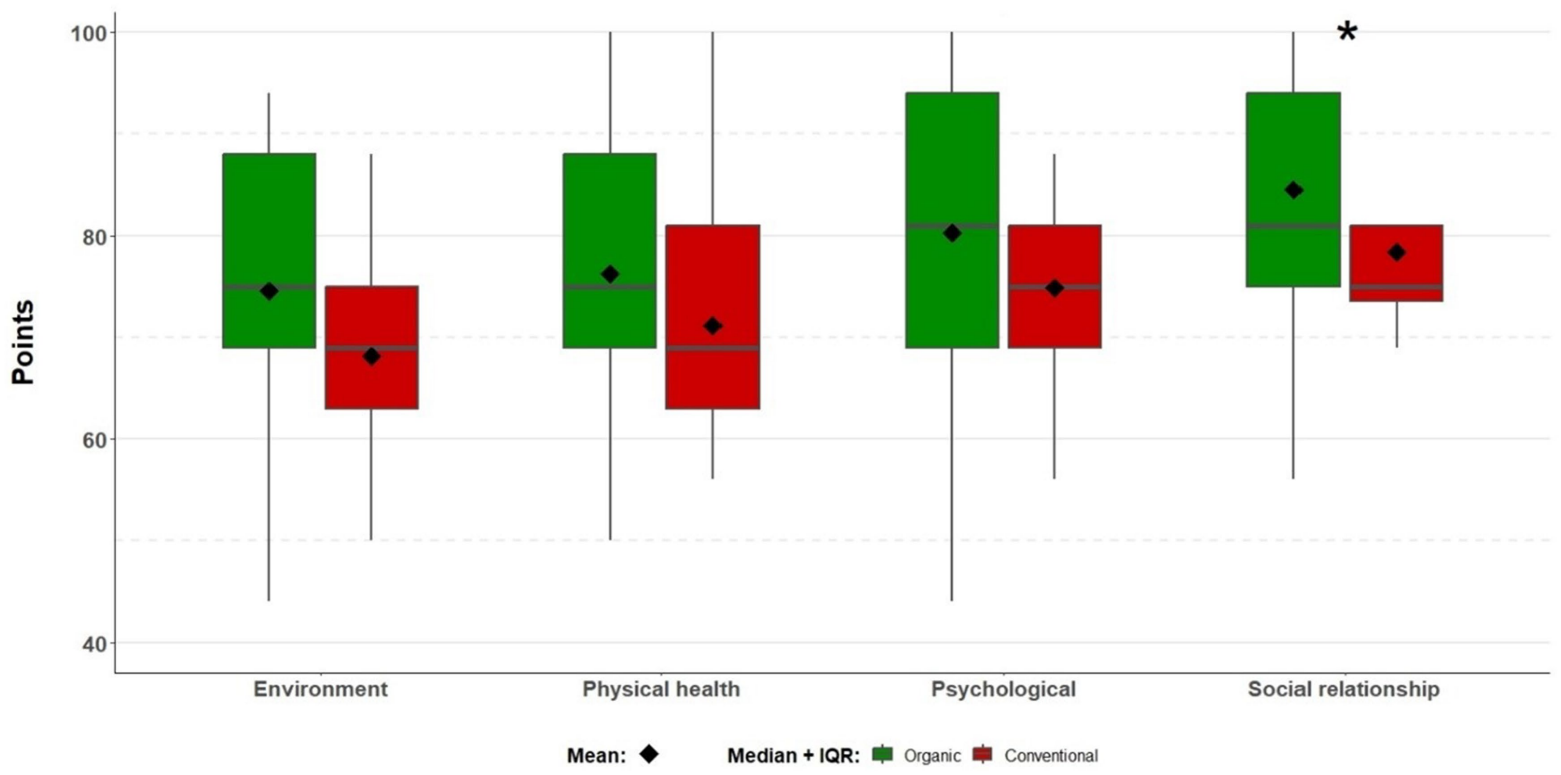
Differences in scores across quality of life domains. * The observed statistically significant difference between the groups (*p* = 0.047).

Analysing the individual domains, it was observed that the study group scored 73.92 ± 14.02 points in the physical health domain, a median of 81 (44–100) points in the psychological domain, a median of 81 (50–100) points in the social relationship domain and a median of 69 (44–94) points in the environmental domain. For the physical health, psychological and ecological domains, no statistically significant differences were observed between the organic and conventional fruit grower groups (*p* > 0.05, U Mann–Whitney test; *p* > 0.05, *t* test), while a statistically significant difference was observed in the social domain (*p* = 0.047, U Mann–Whitney test). No statistically significant differences were observed between the group of fruit growers and their partners (*p* > 0.05, U Mann–Whitney test; *p* > 0.05, *t*-test). The differences in the number of scores in each domain are shown in the graph (Figure 3).

No correlation was found between total quality of life, its domains, and the length of farming, organic agriculture, or organic certification (*p* > 0.05, Spearman test and Pearson test). There was also no correlation between quality of life and body mass, BMI, and age (*p* > 0.05, Spearman test and Pearson test). However, a correlation was found between body height and the Physical Health domain (*p* = 0.17, *r* = 0.325, Pearson test).

### 3.4 Correlations

A correlation was found between subjective perceptions of health status and self-rated health status compared to peers (*p* = 0.005, rho = 0.380, Spearman test), as well as the number of chronic diseases a person had (*p* < 0.001, rho = −0.485, Spearman test). Self-rated health status compared to peers was also correlated with the number of scores obtained in the physical health domain (*p* = 0.006, rho = 0.424, Spearman test), the psychological domain (*p* = 0.018, rho = 0.374, Spearman test), but not in the social relationship and environmental domains (*p* > 0.05, Spearman test). Individuals failing to indicate their health status were also excluded from observations of these relationships. The number of chronic diseases was in turn correlated with the number of points obtained in the physical health (*p* = 0.017, *r* = −0.325, Pearson test) and social relationship (*p* = 0.032, rho = −0.295) domains. Summary scores indicating quality of life were correlated with both self-rated health status compared to peers (*p* = 0.018, rho = 0.371, Spearman test) and number of chronic diseases of study participants (*p* = 0.02, rho = −0.318). There was no correlation between the educational level and financial situation of the respondents and the other parameters related to quality of life and health status (*p* > 0.05, Spearman test).

## 4 Discussion

To our knowledge, this is the first study of its kind to describe the quality of life in a group of fruit growers. It is also the first study to compare the quality of life between a group of producers (fruit growers) of organic and conventional food. This study, therefore, makes a valuable contribution to the development of knowledge about quality of life in the rural population and its possible determinants - the ways of cultivating the orchard.

The overall quality of life in the study group was satisfactory. The median total quality of life score of 300 points (231–388 points) represents 75% of the total possible points. To our knowledge, there are no official specific cut-off points for determining what is a good or poor quality of life. Skevington et al. (35) proposed a score of 50 as the cut-off point between good and poor quality in each domain. Silva et al. (36) proposed a cut-off point of 60 points in their paper. The second study, however, was conducted on people over 60 years of age, so the participants in our group were mainly much younger. The cut-off point of 50 points in the individual domains, on the other hand, was derived from a population with an average age of 45 years, which is comparable to that of the participants in the present study. However, regardless of the criteria used, the results obtained with our study group suggest a good quality of life, both overall and in individual domains. To date, there are no studies on the quality of life among fruit growers. There was, however, research with Polish farmers. A study by Wojewódzka-Wiewiórkowska et al. (37) assessing quality of life of Polish farmers in three aspects (mental comfort, economic situation, and living conditions, including self-assessed health) showed that the financial situation positively (and significantly) influences living conditions, and a positive correlation between living conditions and health was also observed. In contrast, the economic crisis was not significantly related to mental well-being (37). However, this research employed a different method (Structural Equation Modelling - SEM) and did not calculate a quality of life index on a point scale. Therefore, it is not possible to directly relate the results obtained in that work to the results obtained in this study. A study by Ćwirlej-Sozańska et al. (38) from rural areas in eastern Poland, which used the same methodology, also found lower quality of life indicators for farmers compared to our study group. However, this study was conducted with older individuals, who may have had a possibly different quality of life. These studies also demonstrated a relationship between quality of life and age, as well as the number of diseases. In our study, such multiple associations were not observed. This may be due to the small size of our group. In groups that are too small, it is definitely harder to show certain relationships that would probably exist with a larger sample. However, this is a pilot study to analyse the need for a larger study and generalising the results to the general population would be going too far. Previous studies on the rural population, similar to ours, generally indicate poorer results in terms of quality of life.

Herrera Sabillon et al. (39) studied the well-being of European farmers using the method of Structural Equation Modelling (SEM). The results showed that around 22% of farmers expressed low satisfaction with their quality of life, 58% reported moderate satisfaction, and around 20% reported high satisfaction. This distribution varies depending on the country and farm size. Overall, a higher percentage of farmers in Ireland and Finland expressed higher levels of satisfaction with their quality of life. In contrast, a higher percentage of farmers in Poland and Greece expressed low satisfaction with their quality of life. The percentage of farmers with low levels of satisfaction decreased with increasing farm size. These results were obtained using the same method as in the study by Ćwirlej-Sozańska et al. (38) and are similar to them – Polish farmers showed relatively low quality of life indicators in both studies. Due to the different methodology, it is difficult to directly compare the results of Herrera Sabillon et al. (39) with ours, but the Polish fruit growers we surveyed generally showed a higher quality of life than Polish farmers.

One of the possible reasons for this is that Polish fruit growers can be economically satisfied as they earn relatively good incomes compared to Polish farmers in general, as they make extensive use of the subsidies available under the agri-environmental programme (PROW 2023–2027), and organic fruit growers receive relatively high subsidies per hectare of production (40).

Research in Brazil on rural populations found comparable but slightly lower quality of life among people who had always lived in rural areas, compared to those who had spent less than half their lives there. These individuals were also less likely to have a better perception of their overall quality of life (41). In comparison, another study conducted on the general population of Brazil found a better quality of life in rural areas than in the general population in that country (42). The aforementioned study on rural populations was conducted on a much larger population and found an association between quality of life and health satisfaction with factors such as age, education level, financial situation or gender (in comparison, in the general population, the Brazilian study found an association between outcomes and gender, age, financial situation, education or health issues). Another study involving the Lithuanian population, which is geographically and culturally similar to the population participating in this study, showed slightly worse results compared to our research, with an additional lower level of quality of life in the rural population compared to the urban population (43). This research was conducted using the WHOQOL-100 questionnaire, a different questionnaire from the one used in this study. However, it is acceptable to compare the results from both questionnaires with each other. Additionally, these studies have shown an association between quality of life and factors such as income and education.

In contrast, the present study revealed an association between self-assessed health status compared to peers and quality of life, as well as between subjective satisfaction with health status and quality of life. An association was also found between the quality of life and the number of chronic diseases experienced by the participants. The relationship obtained is therefore consistent with the trend observed in studies by other authors, indicating that health status is one of the main determinants of quality of life. Similar observations were made in the research of Głowicka-Wołoszyn et al. (44). This study examined 730 farming families throughout Poland and found that, in each of the separate quality of life classes, farmer households attached the highest importance to health and the quality of the environment in their place of residence, and the lowest importance to education, free time, and social relations. The conducted research confirmed that farmers’ households face a worse demographic, social, and economic situation compared to other socio-economic groups. Unfortunately, the study did not compare the quality of life of farmers with that of other social groups (44). It should be emphasised, however, that these studies employed an entirely different method of assessing quality of life than our studies, which makes it difficult to compare the results directly with those obtained in our studies. The demonstration of only this relationship underlines its relevance. However, the presence of this relationship does not exclude the possibility of links between quality of life and other parameters. There is a need to conduct a similar study on a much larger group of participants to confirm the relationships found and to demonstrate other links between quality of life and other determinants.

The way organic orcharding is carried out is undoubtedly different from conventional orcharding. This is reflected in the various elements of life, which then contribute to its quality. Reducing the use of pesticides can have a positive impact on both health and the environment. More creative solutions, as alternatives to conventional cultivation methods, represent a care for cognitive functions and psychological aspects. More engaging work, on the other hand, can result in reduced leisure time and social contacts. The way in which an orchard farm is run can therefore translate directly into the quality of life of fruit growers. And this is also the case in this study - organic fruit growers scored better in terms of quality of life compared to conventional fruit growers. Similar conclusions were reached in the work of Alvarez-Esteban et al., who demonstrated that organic farms offer a significantly better quality of life and working environment than conventional ones (45). Additionally, Mzoughi (46) showed a correlation between being organic and life satisfaction. Additionally, the paper notes that farmers may adopt ecologically friendly practices to increase their potential for life satisfaction (44). Also, Jansen (47) suggested in his work that organic farm workers may have higher life and job satisfaction. It can therefore be assumed that factors present in the cultivation of organic food are absent in the cultivation of conventional food, which affects the overall quality of life.

When analysing the individual components of quality of life, it should be noted that the best results were obtained in the psychological and social relationship domains, both in the total study group (median 81 points) and among organic (median 81 points) and conventional fruit growers (median 75 points). The worst results were obtained in the environmental domain (median 69 points for the total study group; 69 points among conventional fruit growers and 75 points among organic fruit growers). The scores obtained in all domains for both conventional and organic fruit growers were above the proposed cut-off points, indicating good quality of life in each domain for both groups. However, a significant difference was shown between the two groups of orchardists in the social relationship domain. Organic fruit growers scored significantly higher in this domain, compared to conventional fruit growers. From the questionnaire design, it is worth noting that this domain encompasses personal relationships, social support, and sexual activity (34). A deeper analysis reveals that statistical significance was primarily demonstrated for personal relationships (*p* = 0.016). This would mean that organic fruit growers would be more satisfied with the relationships they form with other people, compared to conventional fruit growers. However, this may be due to the way the study was recruited. As research by Mellor et al. (48) has demonstrated, the need to belong has a significant impact on subjective well-being, which is partly attributed to feelings of loneliness arising from an unmet need for belonging. In the present study, organic orchardists were recruited based on their membership in an association of organic orchardists. They were also overwhelmingly living in marriages. Thus, both factors undoubtedly contributed to at least a partial satisfaction of the need for belonging, thereby raising the level of satisfaction with personal relationships as well as life as a whole. On the other hand, the higher satisfaction with personal relationships among organic fruit growers may be due to more frequent contacts with other organic fruit growers to exchange experiences and insights - for, as indicated in the introduction, the system of organic farming and fruit growing often requires a hefty dose of creativity and openness, which certainly influences the ease of establishing contacts and exerts a need to maintain them. In their study, Masambuka-Kanchewa et al. (49) found that organic farmers were more likely to communicate directly with consumers, but also used various means of mass communication, including social media. The lack of communication from conventional farmers was attributed to their busy work schedules on the farm, which left them with little time to spare. In contrast, organic farmers were more open to communication, a trait rooted in their beliefs and values regarding sustainable farming practices and environmental conservation (49). Organic farmers and fruit growers may therefore be more open to dialogue and communication, which can improve their satisfaction with the relationships they have created. However, there is not enough data from the survey to unequivocally state the reason for this difference.

However, the lack of significant dependencies in the psychological domain between fruit growers from the organic and conventional groups is surprising. Several studies have directly indicated or suggested that organic farmers experience better psychological well-being compared to conventional farmers. In the research of Yazd et al. (50), the authors found that the method of agricultural management has a significant impact on the psychological well-being of both owners and agricultural workers, with most studies to date pointing to higher indicators of well-being and quality of life of people working in organic farms than in conventional ones. In turn, another study by the same authors (51) found that crop irrigators working in organic gardening in Australia presented a lower level of mental stress than their colleagues in the conventional sector. The results suggest that farmers with substantial financial capital, adequate productivity, and sound land management cope better with weather uncertainty and are more resilient to it (51). Ultimately, an extensive review of 29 different studies revealed that organic farmers experienced better mental and physical health globally compared to conventional farmers. Furthermore, the factors correlated with farmers’ health tended to be psychological, social, financial and agricultural (52). It would therefore be expected that similar results would be obtained in this study, especially in the psychological domain. The lack of such dependencies could be due to a temporary situation or a small group. However, the observed results of other authors encourage further research in this area, especially in the development of agricultural models.

The physical health domain was correlated with self-rated health in relation to the health of peers and with the prevalence of chronic disease within the study group. This domain encompasses factors such as pain sensations, work restrictions, and medication use, among others. It is therefore natural that increasing numbers of illnesses and poorer health status will be associated with poorer scores on this domain.

The social relationships domain, on the other hand, was inversely correlated with the amount of chronic illness among participants. The orchardists who were less ill were therefore more satisfied with their social relationships. This result can be justified by the difficulties in establishing and maintaining relationships that some diseases bring with them. Chronic conditions often require regular medication or treatment, which can significantly impact the quality of social relationships.

A correlation was also found between self-rated health and scores in the psychological domain compared to peers. The issues of poorer health and distress, along with their links, have been the subject of many studies, as presented in the meta-analysis by Barry et al. (53). Naturally, poorer health will translate into poorer psychological well-being and, consequently, a lower quality of life in this domain. On the other hand, poor mental health will impact health and cause health complications. The meta-analysis mentioned above also observed an increased risk of mortality among people reporting high compared to low levels of distress. Taking care, therefore, of health-related issues and the treatment of existing illnesses in the study group will be crucial to improve both the overall quality of life and the quality expressed in the individual domains: physical health, psychological and social relationships.

### 4.1 Limitations

Our study has several limitations that should be taken into account when interpreting the results. Firstly, as indicated, this study involved a relatively small group. A study conducted on such a small group of individuals may have resulted in some of the relationships commonly observed in wider populations not being demonstrated in this group. However, the present study is a pilot study to verify the need for this type of research on this group, rather than to generalise the results. Secondly, the recruitment method may have influenced some of the results, particularly in the quality of life domain related to social relationships. A different approach to recruitment, which does not presuppose membership in various groups (e.g., associations), is definitely more advisable in this respect. An example of such recruitment is the random selection of participants based on issued certificates for the organic food label, followed by the identification of the nearest groups growing conventional food. Thirdly, one region of the study (Mazowieckie and Łódzkie Voivodeships, central Poland) is limited to specific environmental conditions. Orchardists operating in other areas of the country may assess certain aspects of their lives differently due to the varying locations and conditions present in those areas. And finally, the survey was conducted in only one repetition. The survey period took place at a time when orchardists were no longer working intensively in their orchards, resulting in a different lifestyle for them. Surveying at least several times throughout the year would have given a better picture of the quality of life among fruit growers. Again, however, this is a pilot study that had other assumptions. These assumptions have been met; from the results, we can see the need for further research with similar groups, taking into account the factors detailed in this paragraph.

To summarise the discussion, the results presented here support our hypothesis that organic fruit growers have a better overall quality of life than conventional fruit growers. The main reason for this result is the higher score for social relationships among organic fruit growers than among conventional ones.

## 5 Conclusion

In conclusion, the study group had a satisfactory quality of life. However, the organic orchardists had a better overall quality of life, including higher quality social relationships. Organic fruit growers also rated their health better in relation to their peers, compared to conventional fruit growers. The quality of life in the study group was closely linked to health status and morbidity within the group. Taking care of this aspect is crucial in improving the quality of life among fruit growers. Further research on the determinants of quality of life should be conducted with fruit growers, with a special focus on the division between organic and conventional fruit growers, in larger groups, using appropriate sampling and multiple measurements.

## Data Availability

The raw data supporting the conclusions will be made available by the authors, without undue reservation.
